# Preservation of the Cadaver

**Published:** 1871-05

**Authors:** 


Preservation of the Cadaver.—According to M. Devergie, of the Paris School of Practical Anatomy, a mixture of three parts glycerin and one of carbolic acid injected into dead bodies, will prevent any unpleasant odor emanating from them for several months.

